# Time out: should vitamin D dosing be based on patient's body mass index (BMI): a prospective controlled study

**DOI:** 10.1017/jns.2021.100

**Published:** 2021-12-13

**Authors:** Mir Sadat-Ali, Khalid W. AlTabash, Haifa A. Al-Turki, Sulaiman A. AlMousa, Hasan N. AlSayed

**Affiliations:** 1 Department of Orthopaedic Surgery, King Fahd Hospital of the University, Imam AbdulRahman Bin Faisal University, P.O. BOX 40071, AlKhobar 31952, Saudi Arabia; 2 Department of Obstetrics and Gynecology, King Fahd Hospital of the University, Imam AbdulRahman Bin Faisal University, AlKhobar 31952, Saudi Arabia

**Keywords:** Body mass index, Deficiency, Dosage, Vitamin D

## Abstract

The recommended daily dose of vitamin D is 2000 IU was found to be insufficient in many patients. The objective of the present study is to find whether the daily dose of vitamin D should be based on BMI. Two hundred and thirty patients with an established vitamin D deficiency (serum level of 25 Hydroxy vitamin D3 (25OHD3) of ≤20 ng/ml) and patients with BMI ≥30 kg/m^2^ were included in the study. Demographic data, comorbidities and BMI were recorded. Pre-treatment and post-treatment serum 25OHD3, calcium, phosphorus and parathyroid hormone (PTH) were tested at 0-, 3- and 6-month periods. Patients were treated with a standard dose of 50 000 IU of vitamin D weekly and 600/1200 mg of calcium a day. Once their level of 25OHD3 reached ≥30 ng/ml, patients were randomised into two groups. Group A received a standard recommended maintenance dose of 2000 IU daily and Group B patients received 125 IU/kg/m^2^ of vitamin D3. The data were entered in the database and analysed. The mean age of Group A was 50⋅74 ± 7⋅64 years compared to 52⋅32 ± 7⋅21 years in Group B. In both groups, pre-treatment vitamin D level was ≤15 ng/ml and increased to 34⋅6 ± 2⋅6 and 33⋅7 ± 2⋅4 ng/ml at the end of 3 months treatment with a dose 50 000 IU of vitamin D3 and calcium 600/1200 mg once a day for group A and group B, respectively. At 6 months, patients in Group A 25OHD3 level was 22⋅8 ± 3⋅80 and in Group B was 34⋅0 ± 1⋅85 ng/ml (*P* < 0⋅001). This preliminary study suggests that obese patients need higher dosage of vitamin D than the recommended dose. It is prudent that the dosage should be based on the BMI to maintain normal levels for a healthy musculoskeletal system.

## Introduction

Vitamin D3 (Cholecalciferol) is a fat-soluble secosteroid that is important for increasing intestinal absorption of calcium, magnesium and phosphate. In the past, it was believed that that low levels of vitamin D caused rickets and osteomalacia, but recently it was recognised that it has a broader influence on skeletal health as vitamin D receptors were found in multiple organs^(1–7)^. It is now believed that low levels of vitamin D influence many disease processes and adequate levels can prevent many diseases. On the other side, concerns were raised due to toxic doses of vitamin D which may cause atherosclerosis^(8)^.

Since the realisation of the importance of vitamin D, a one-size-fits-all dosing strategy was utilised for adult patient with the only exception being in paediatric patients in which dosing was based on patients’ characters. Initially, the daily requirement dosage of vitamin D3 was recommended as 400–800 IU/d. However, this was repeatedly adjusted to reach a daily dose of 2000 IU and even reaching up to 4000 IU in recent references^(9–11)^. All recommendations lacked any scientific basis as one dose was suggested irrespective of age, sex and BMI. This suggests that the correct daily requirement dosage of vitamin D remains poorly defined.

Recently, after an extensive review, Ghanaati *et al.*^(12)^ recommended that a dose of 5000 IU/d is required to maintain a healthy life. There is no consensus in the literature regarding what should be the ideal daily dosage. Observation studies have shown that the old concept of one dose fits all may not be optimum in different genders and people with different body morphology. The question why a one dose for all was not sufficient is due to the fact as some of the patient's levels do not increase to the intended levels, hence different researchers proposed different doses. Many studies and in our own clinical experience, we found that obese patients do not respond to the regular recommended doses leaving them vulnerable to the after effects of vitamin D deficiency. The present study was carried out to compare the post-treatment serum levels of vitamin D after a standard maintenance dose of 2000 IU of vitamin D3 and a dose of 125 IU/kg/m^2^ daily for 3 months.

## Patients and methods

Two hundred and thirty patients with an established vitamin D deficiency with BMI ≥30 kg/m^2^ were included in the study and followed for 6 months. All women who were included in the present study were treated at orthopaedic clinics for osteoarthritis and osteoporosis and obstetrics and gynaecology clinics. This study was conducted according to the guidelines laid down in the Declaration of Helsinki and the study was approved by the Institutional Review Board of the Imam AbdulRahman Bin Faisal University, Dammam vide 2114/2019. An informed written consent was obtained from all patients.

Demographic data and weight and height was taken to calculate BMI. Pre-treatment and post-treatment serum 25OHD, calcium, Phosphorus, PTH were done at 0, 3 and 6 months. Deficiency was defined as serum level of 25OHD of ≤20 ng/ml, insufficiency 21–29 and ≥30 ng/ml as normal. Patients were treated a standard dose of 50 000 IU vitamin D weekly and calcium 600 mg once a day for 3 months. Once the level of 25OHD3 reached ≥30 ng/ml, patients were divided into two groups for further study. Group A received a standard recommended maintenance dose of 2000 IU daily and Group B patients received 125 IU/kg/m^2^ of vitamin D3. Blood analysis were carried out in house. 25OHD3 level was measured using Chemiluminescence immunoassay using DiaSorin (Liaison®, Saluggia, Italy) analyzer.

### Statistical analysis

Results are expressed as mean values and standard deviation. The effect of the treatment between groups was analysed by paired, two-sided *t*-tests. Multivariate regression analysis was performed to identify the variables to age, BMI, cholecalciferol dose in IU and fixed dose. A *P*-value < 0⋅05 was considered to be statistically significant.

## Results

**Fig. 1.**
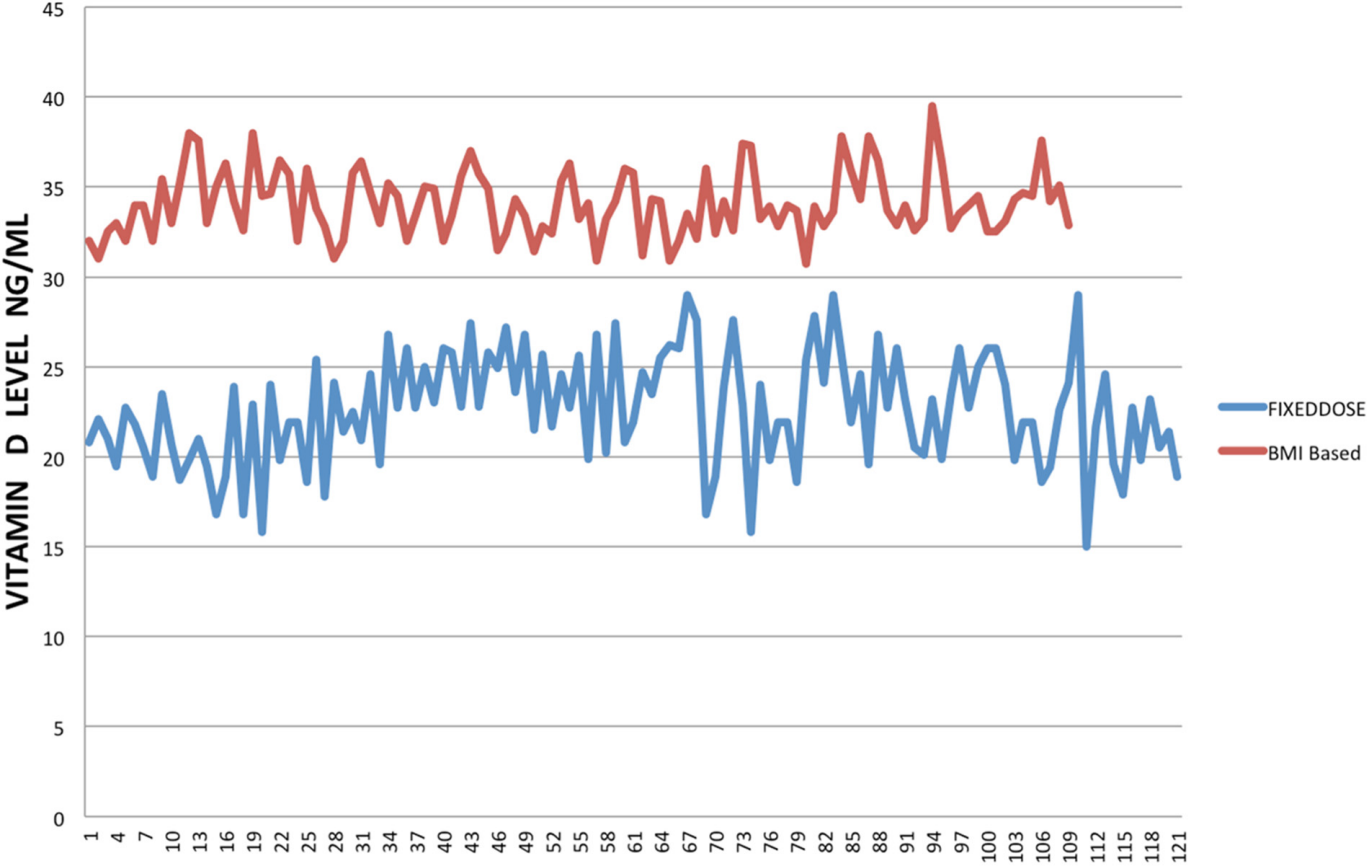
Comparison of 25OHD levels between the dosage based on BMI and a fixed dose of 2000 IU daily.

Two hundred and thirty patients were in the study group and were followed for 6 months. The classification of BMI is given in Table 1. The demographic data is given in Table 2. The mean age of Group A was 50 ± 7 years, compared with Group B was 52 ± 7 years. Pre-treatment vitamin D level was 14 ± 3 *v*. 14 ± 3 ng/ml which increased to 34 ± 2 and 33 ± 2 ng/ml at the end of 3 months with a dose 50 000 IU of vitamin D3 and calcium 600/1200 mg once a day (Table 2). At 6 months, patients in Group A 25OHD3 level 22 ± 3 and in Group B was 34 ± 1 ng/ml (*P* < 0⋅001), 95 % CI 10⋅4098 to 11⋅9902 PTH level was 8 ± 1 *v.* 6 ± 1 pc/ml (*P* < 0⋅001, 95 % CI −2⋅4307 to −1⋅6293) (Table 3). Fig. 1 gives the graphic picture of comparison of level between the flexible dose and fixed dose. In patients of fixed dose, the starting level which was 34 ± 1 ng/ml at the start of with a fixed dose of 2000 IU daily dropped to 22 ± 3 ng/ml (*P* < 0⋅0001) at the 3-month interval. Patients in the flexible dose required an average of 3984 ± 160 IU to maintain the level to 34 ± 1 ng/ml of 25OHD3.

**Table 1.** Classification of body mass index

Category	Body mass index (kg/m^2^)
Normal Weight	18–24
Overweight	25–29
Obese (Class I)	30–34
Obese (Class II)	35–39
Obese (Class III)	≥40

**Table 2.** Demographic data of all enrolled patients

Number of Patients	230
Age (Years)	51 ± 7
Diabetic Mellitus	129
Height in Centimetres	152 ± 4
Weight in Kilograms	97 ± 3
Serum Calcium Level (mg/dl)	9 ± 0⋅3
Parathormone (pc/ml)	9 ± 1
Serum 25OHD3 (ng/ml)	14 ± 3

**Table 3.** Comparison between the groups after 6 months

Parameters	Group A (2000 IU)	Group B (125 IU/kg/m^2^)	*P*-value
Number of patients	121	109	
Age (Years)	51 ± 8	52 ± 7	0⋅1
BMI (kg/m^2^)	31 ± 1	32 ± 1	0⋅5
3-month Vitamin D Level (ng/ml)	34 ± 2	33 ± 2	0⋅3
6-month Vitamin D Level (ng/ml)	22 ± 3	34 ± 1	0⋅001
6-month Parathormone (pc/l)	8 ± 1	6 ± 1	0⋅001

## Discussion

Our study shows that a daily fixed dose of vitamin D3 was not enough to maintain the serum level of 25OHD ≥30 ng/ml. We observed that patients with higher BMI required a much larger dose of vitamin D indicating that the dosage of vitamin D should be based on the BMI. The analysis suggests that persons who are overweight and taking a recommended fixed dose of 2000 IU of vitamin D were at substantially higher risk of deficiency/insufficiency which may lead to osteomalacia, bone pain and muscle weakness.

Why vitamin D is low in obese subjects? Various theories have been suggested and it appears that this may be the result of several processes one of them is as vitamin D being a fat-soluble vitamin is stored in the adipose tissue and in the obese subjects larger the storage, hence large quantity is sequestrated in the adipose tissue with less amount of circulating 25OHD^(13)^.

Low dietary intakes of vitamin D in obese individuals was also raised as a cause but this was dispelled by the study of Walsh *et al.*^(14)^ as they found dietary intake of vitamin D in obese and normal weight persons was quite similar dispelling the earlier dietary issues. The Endocrine Society guidelines suggested that in obese people the dose of vitamin D to maintain normal vitamin D levels require three times more vitamin D dosage than the lean individuals^(15)^. It was later reported that individuals with BMI ≥30 kg/m^2^ achieved 20–30 % less 25OHD level than the subjects of ≤25 kg/m^2^ when same dose of vitamin D was given^(16,17)^. To raise the serum level of 25OHD to reach normal levels in obese subjects, a formula was needed by which a dose can be given based on the BMI and our study was based on giving a dose of 125 IU/kg/m^2^. Our results raise the contemplation that treatment for vitamin D deficiency should be based on BMI which can increase the levels to return to normal levels and improve musculoskeletal health.

A one-size-fits-all dosing practice is good enough for many drugs, but several important drugs like antibiotics, chemotherapeutic agents and hormonal replacement, heparin, digoxin, phenytoin and monoclonal antibodies, to name few, are all given on weight-based dosing^(18)^. Furthermore, it has been suggested that in obese individuals, oral contraceptives do not work well as fixed dosage and that fixed dosage is not enough to prevent conception, putting women at risk of an unwanted pregnancy^(19)^. As of now, we recognise that vitamin D plays an important role not only in musculoskeletal health but also in prevention of many diseases myocardial infarction^(20)^, prevention of adult onset diabetes and pre-diabetes^(21)^ and prevention of cancer and cardiovascular disease^(22)^ and still many over weight and obese patients are not getting optimal dose which raise the 25OHD levels to maintain good health. Secondly, absorption and metabolism is different in obese, overweight and normal weight patients. We believe that time has come where vitamin D should be prescribed based on the BMI which will provide adequate levels of 25OHD. Moreover, it has been speculated that in the US by 2030 nearly 50 % of the population will be classified as obese and 25 % severely obese and in Australia, the percentage is as high as 65 %^(23,24)^. If these obese individuals were given a fixed dose of vitamin D, then we are risking them of multiple complications due to the deficiency/insufficiency. With the recent revelations of the link between vitamin D and many diseases^(25)^, including Covid-19^(26–28)^ it will be detrimental to leave the one section of the population to the effects of the vitamin D at suboptimal levels. It is time now to be prudent and change the dosing practices which will help the obese population, in particular, to remain healthy.

### Limitations

The present study has few limitations. Firstly, our patient numbers are small and, secondly, we have studied only female population, and lastly, we used an arbitrary dose of 125 IU/kg/m^2^ was used. We have to perform randomised trials of interventions to reduce low levels of vitamin D with different dosage of 100 IU, 110 IU and 150 IU/kg/m^2^ for which we need to have large population of obese subjects.

## Conclusion

The findings of the present study clearly highlight that vitamin D supplementation should be on a flexible dose of 125 IU/kg/m^2^ to maintain the optimum level of 25OHD for a healthy life. As other important drugs are given on a BMI basis, vitamin D should also be added to that list so that overweight and obese patients who are at more risk of common diseases should not suffer the affects of low vitamin D levels, till that period further studies confirm alternate ways to keep the serum 25OHD levels higher than normal levels.
